# Fulvic acid foliar application: a novel approach enhancing antioxidant capacity and nutritional quality of pistachio (*Pistacia vera* L.)

**DOI:** 10.1186/s12870-024-04974-0

**Published:** 2024-04-04

**Authors:** Mohammadali Nikoogoftar-Sedghi, Vali Rabiei, Farhang Razavi, Sanaz Molaei, Ali Khadivi

**Affiliations:** 1 Department of Horticulture, Faculty of Agriculture, University of Zanjan, Zanjan, Iran; 2 Department of Horticultural Sciences, Faculty of Agriculture and Natural Resources, Arak University, Arak, 38156-8-8349 Iran

**Keywords:** Antioxidant capacity, Fulvic acid, Pistachio, Quality attributes

## Abstract

**Background:**

The global growth of pistachio production has prompted exploration into sustainable agricultural practices, on the application of humic substances such as fulvic acid in enhancing the quality of horticultural crops. The present study was carried out in Qom province, Iran, on 20 years old pistachio (*Pistacia vera* L. cv. Kaleh-Ghoochi) trees and investigated the impact of foliar spraying of fulvic acid at varying concentrations (1.5, 3, and 4.5 g L^− 1^) on the antioxidant and quality properties of pistachio. The different concentrations of fulvic acid were applied at two key stages: at the initiation of pistachio kernel formation (late June) and the development stage of pistachio kernel (late August), as well as at both time points. Following harvest at the horticulturally mature phase, various parameters, including total phenols, flavonoids, soluble proteins, soluble carbohydrate content, antioxidant capacity, and antioxidant enzyme activity, were assessed.

**Results:**

Results indicated that foliar application of fulvic acid, particularly at 1.5 g L^− 1^ during both late June and August, effectively increased phenolic compounds (31.8%) and flavonoid content (24.53%). Additionally, this treatment also augmented antioxidant capacity and heightened the activity of catalase (CAT) (37.56%), ascorbate peroxidase (APX) (63.86%), and superoxide dismutase (SOD) (76.45%). Conversely, peroxidase (POX) (41.54%) activity was reduced in fulvic acid-treated nuts compared with controls. Moreover, the content of chlorophyll (45%) and carotenoids (46.7%) was enhanced using this organic fertilizer. In terms of mineral elements, the increment was observed in zinc (Zn) (58.23%) and potassium (K) (28.12%) amounts in treated nuts. Additionally, foliar application of fulvic acid led to elevated levels of soluble carbohydrates and proteins in treated nuts.

**Conclusions:**

In the present study, application of fulvic acid resulted in enhancement of antioxidant activity and quality traits of pistachio nut through an increase in total phenol, flavonoids, chlorophyll, carotenoids, K, Zn, and also activity of antioxidant enzymes. Therefore, use of fulvic acid emerges as a promising strategy to enhance the quality and nutritional attributes of pistachios, contributing to sustainable agricultural practices and improved crop outcomes.

## Introduction

Pistachio (*Pistacia vera* L.) holds significant economic importance as a producer of edible nuts within the Anacardiaceae family, renowned for its nutritional richness [1, 2]. The pistachio kernel, consumed in various forms such as dried, fresh, and salted roasted, is valued for its high protein content, unsaturated fatty acids (linolenic, linoleic, and oleic acid), carbohydrates, essential elements (calcium, potassium, iron, phosphorus, and copper), vitamins, dietary fibers, phenolic compounds, and natural antioxidants (carotenoids, anthocyanin, flavonoids, and phytoestrogens) [3, 4]. Originating from the Asia Minor region, including countries such as Iran, Turkey, Lebanon, Syria, and Afghanistan, today, major global producers of pistachio nuts include Iran, the USA, Syria, and Turkey [2, 5]. Iran, particularly recognized for housing over 70 genotypes and cultivars of female and male pistachios, cultivates well-known cultivars, such as ‘Ohadi’, ‘Kaleh-Ghoochi’, ‘Badami’, ‘Ahmad-Aghaei’, ‘Momtaz’, and ‘Rezaei’. The ‘Kaleh-Ghoochi’ cultivar, celebrated for its light green, round-shaped kernels, stands out due to its large fruit size, high yield, and split percentage [1, 2, 6]. Despite the global prominence of pistachios, challenges arise in postharvest handling, with only 10% of the total production consumed in its fresh form in Iran, and about one-third of harvested pistachios incurring losses during shelf life and distribution stages [7, 8]. Factors such as microbial contamination and biochemical and physiological changes contribute to diminished shelf life and quality, leading to increased microbial spoilage, weight loss, alterations in sensory attributes, surface color, and nutritional value, and ultimately compromising the marketability of the fresh fruit [8, 9]. To counteract these detrimental changes, various pre and post-harvest treatments have been explored, including the application of natural, edible, and environmentally friendly chemicals and coatings, along with appropriate storage conditions [10].

Humic substances, encompassing humus, fulvic acid, humic acid, humate, and humin, play a pivotal role in plant nutrition and soil fertility. The application of these substances as soil fertilizers or foliar sprays has been demonstrated to enhance plant health, stress resistance, yield, and post-harvest nutritional value across diverse plant species. Fulvic acid, a compound comprising aromatic and aliphatic organic acids, exhibits high water solubility across various pH conditions. Its chemical reactivity is attributed to a high oxygen content, carboxyl groups, and hydroxyl groups. Notably, fulvic acid possesses a lower molecular weight and smaller size than humic acid, facilitating absorption through leaves, stems, and roots of plants. As a foliar spray, fulvic acid promotes the transport of trace minerals from the plant surface to metabolic sites within plant tissues, thereby enhancing overall plant productivity. Its chelating properties, coupled with attributes such as plant compatibility, non-toxicity, and effectiveness at low concentrations, make fulvic acid a valuable agricultural tool [11, 12].

Previous studies have highlighted the positive impact of foliar spray applications of fulvic acid, including increased plant biomass, antioxidant enzyme activity, and photosynthetic pigments in wheat [11]. Additionally, the exogenous application of fulvic acid has been shown to enhance proline content and the activity of antioxidant enzymes (SOD, POD, and CAT) in maize plants under drought stress [13]. Fulvic acid has also demonstrated its potential to enhance fruit quality and yield in tomato plants when applied as a foliar spray. Furthermore, in Damask rose, the use of fulvic acid, along with ginger extract as a foliar spray, resulted in increased content of total phenols, anthocyanin, carotenoids, and essential elements such as Fe, Zn, Mg, N, P, and K [14].

Despite the existing evidence on the impact of fulvic acid application on the postharvest quality of various crops, there is a notable absence of information regarding its effects on the nutritional value of pistachios. Consequently, the present study aimed to investigate the impact of fulvic acid application in different concentrations and at various stages of kernel development on the biochemical, physicochemical, and sensory attributes of pistachio (*P. vera* cv. Kaleh-Ghoochi) fruit.

## Materials and methods

The research was conducted in 2021 at a commercial orchard located in the northwest region of Qom province (34˚54’16’’N and 50˚57’46’’E), Iran, characterized by a semi-arid climate, an average annual rainfall of 136 mm, and an average annual temperature of 19˚C. The selected pistachio cultivar was ‘Kaleh-Ghoochi’ grafted on Badami-Riz rootstock, and the trees (36 trees) had an age of 20 years and were cultivated at a distance of 7*2 m. Healthy trees exhibiting uniform growth of the ‘Kaleh-Ghoochi’ were chosen for the study. The chemical and physical traits of soil are shown in Table 1.

The fulvic acid which was prepared from Sigma Aldrich Co, USA, was foliar applied at concentrations of 1.5, 3, and 4.5 g L^− 1^, and distilled water served as the control. The application was conducted at two distinct time points: (1) at the onset of pistachio nut (kernel) formation in late June and (2) at the stage of full development of pistachio kernel at the end of August. Moreover, a group of trees was foliar sprayed at both end of June and August. It should be mentioned that the whole trees were sprayed with foliar spray. Standard nutritional, irrigation, and orchard management practices were employed following previous years’ calendars. Drip irrigation was administered every 10 days (6000 m^3^ ha^− 1^), and pistachio nuts were harvested at the horticultural maturity phase, and promptly transferred to the physiology laboratory.

**Table 1:** The physical and chemical characteristics of the soil studied

Soil depth (cm)	Organic matter (%)	pH	EC (ds m^− 1^)	K (mg K^− 1^)	P (mg K^− 1^)	N (%)	Soil texture	Sand (%)	Clay (%)	Silt (%)	Organic carbon (%)
40	1.33	7.7	3.2	508	32	0.07	Loamy	51	42	7	0.8
80	1.7	7.8	3.8	520	32	0.09	Loamy	51	42	7	1

### Total phenol and flavonoid content

For extract preparation, 1 g of kernel tissue was homogenized in 80% methanol and centrifuged at 6000 g for 10 min. The total phenol content was determined by mixing 0.1 ml of the obtained extract with 2 ml of 7% sodium carbonate and 0.1 ml of Folin-Ciocalteu reagent. Absorbance was recorded at 720 nm [15]. Flavonoid content was measured using a colorimetric method, wherein 0.25 ml of the extract was mixed with sodium nitrite, aluminum chloride, and sodium chloride, and the absorbance was recorded at 507 nm [16].

### Antioxidant capacity

Antioxidant activity was assessed using the DPPH assay following the method of Dehghan and Khoshkam [17]. Methanolic extract was added to DPPH solution, and the inhibition of DPPH radical activity was calculated as a percentage using the formula:

The DPPH activity (% inhibition) = [(Abs control - Abs sample) / Abs control] ×100.

### Antioxidant enzymes

Enzymatic extracts were prepared by grinding 1 g of kernel tissue with phosphate buffer containing 1% polyvinylpyrrolidone (PVP) and ethylenediaminetetraacetic acid (EDTA). The obtained extract was centrifuged at 12,000 g and 4 °C for 20 min and then the supernatant was used as an enzyme extract to essay the activity of antioxidant enzymes.

### CAT activity

Catalase (CAT) activity was measured by adding 100 µl enzyme extract to potassium phosphate buffer, and hydrogen peroxide, and recording the reduction absorbance at 240 nm for 1 min [18].

### APX activity

Ascorbate peroxidase (APX) activity was determined by mixing 100 µl enzyme extract with H2O2, potassium phosphate buffer, and ascorbate. The reduction absorbance was recorded at 290 nm for 1 min and indicated as a unit per mg protein [19].

### POX activity

The activity of POX was assayed by guaiacol and the method of Plewa et al. [20]. The assay mixture included 30 µl guaiacol (1%), 300 µl H_2_O_2_(3%), 2650 µl potassium phosphate buffer (pH = 7, 50 mM), and 20 µl enzyme extract. The absorbance was detected at 470 nm in 1 min and the activity of POX was expressed as unit per mg protein min^− 1^.

### SOD activity

The method of Zhang et al. [21] was used to measure the SOD activity. 50 µl enzyme extract was added to the assay mixture that contained sodium phosphate buffer (pH = 6.8, 25 mM), methionine (13 mM), EDTA (0.1 mM), nitroblue tetrazolium (75 µM), riboflavin (75 µM). After the mixture was exposed to the fluorescent light for 20 min, the absorbance was recorded at 560 nm.

### Total chlorophyll and carotenoid content

To estimate total chlorophyll and carotenoid content, 1 g of fresh kernel tissue was homogenized in 10 ml of acetone 80% using Ultra-Turrax homogenizer and then centrifuged at 4500 g at 4 °C for 10 min. The absorbance was measured at 652, 510, and 480 nm and the content of total chlorophyll and carotenoid was calculated by following formulas [22]:

Total Chlorophyll (mg g^− 1^ FW) = (D625*1000/34.5) * (V/1000*W).

Carotenoids (mg g^− 1^ FW) = (7.6(D480-1.49*D510)) * (V/1000*W).

D: The number read by the spectrophotometer.

V: the volume of acetone.

W: The weight of the sample.

### Determination of mineral elements

K and Zn amounts were measured by drying pistachio kernels, converting them to ash, digesting the ash with hydrochloric acid, and assessing mineral content using a flame photometer and atomic absorption spectrophotometry, respectively [23].

### Soluble carbohydrates content

Ground pistachio kernel (0.5 g) was homogenized with 70% ethanol, and centrifuged at 4500 g for 15 min. Then, 100 µl of supernatant was added to anthrone (pure anthrone plus sulfuric acid 72%) and was placed in the water bath for 10 min. Eventually, the absorbance was detected at 625 nm, and pure glucose was used as standard [24].

### Soluble protein content

Protein content was estimated by homogenizing 1 g of the kernel with Tris-HCl, centrifuging, and using the supernatant for the Bradford assay. Absorbance was recorded at 595 nm, and protein content was calculated using a standard curve [25].

### Statistical analysis

The study employed a randomized complete block design with factorial treatments in three replications, each consisting of three trees. Statistical analysis was performed using analysis of variance (ANOVA) with SAS software (version 9.3), and differences were evaluated using Duncan’s multiple range test at a significance 0.05 level.

## Results

The variance analysis of obtained data demonstrated that use of fulvic acid had a significant impact on all evaluated traits except total flavonoid content of the pistachio kernel. On the other, the time of fulvic acid foliar spray had a significant effect on total phenols, flavonoid, Zn, and soluble protein content as well as antioxidant enzyme activity. Moreover, the interaction of fulvic acid application time and treatment concentration had a significant impact on all studied characteristics except in total phenol, chlorophyll, K, and soluble carbohydrate content (Table 2).

**Table 2:** The results of variance analysis of the impact of fulvic acid treatment on biochemical characteristics of pistachio ‘Kaleh-Goochi’

						Mean square								
Source	Df	Total phenol	Total flavonoid	Antioxidant capacity	CAT	POX	APX	SOD	Chlorophyll content	Carotenoid content	K	Zn	Soluble carbohydrate	Soluble protein
Block	2	138.56^ns^	37.52^ns^	83.42^ns^	0.16^ns^	0.04^ns^	54.48^ns^	2271.54^ns^	0.48^ns^	0.003^ns^	0.02^ns^	73.63^ns^	16.88^ns^	8.17^*^
Time	2	2467.77^**^	226.08^*^	32.67^ns^	16.34^**^	0.21^*^	107.94^*^	12.175.06^**^	0.04^ns^	0.007^ns^	0.01^ns^	481.19^**^	22.21^ns^	7.12^**^
Concentration	3	2067.8^*^	73.38^ns^	530.2^**^	90.04^**^	0.78^**^	521.37^**^	36020.66^**^	0.47^*^	0.21^**^	0.14^**^	2673.34^**^	165.77^**^	25.02^**^
Time × Concentration	6	420.78^ns^	142.85^*^	171.49^*^	11.86^**^	0.28^**^	97.71^*^	5935.46^**^	0.04^ns^	0.02^**^	0.28^ns^	583.6^**^	16.08^ns^	7.31^**^
Error	22	231.5	41.01	60.82	2.29	0.04	31.01	1109.11	0.11	0.005	0.01	31.96	14.82	1.16
CV (%)		10.42	6.49	11.45	15.5	25.1	20.51	23.54	12.56	10.75	12.09	19.7	15.66	9.51

ns, *, and **: non-significant, significant at *P* ≤ 0.05, and significant at *P* ≤ 0.01, respectively

### Total phenol and flavonoid content

**Fig. 1:**
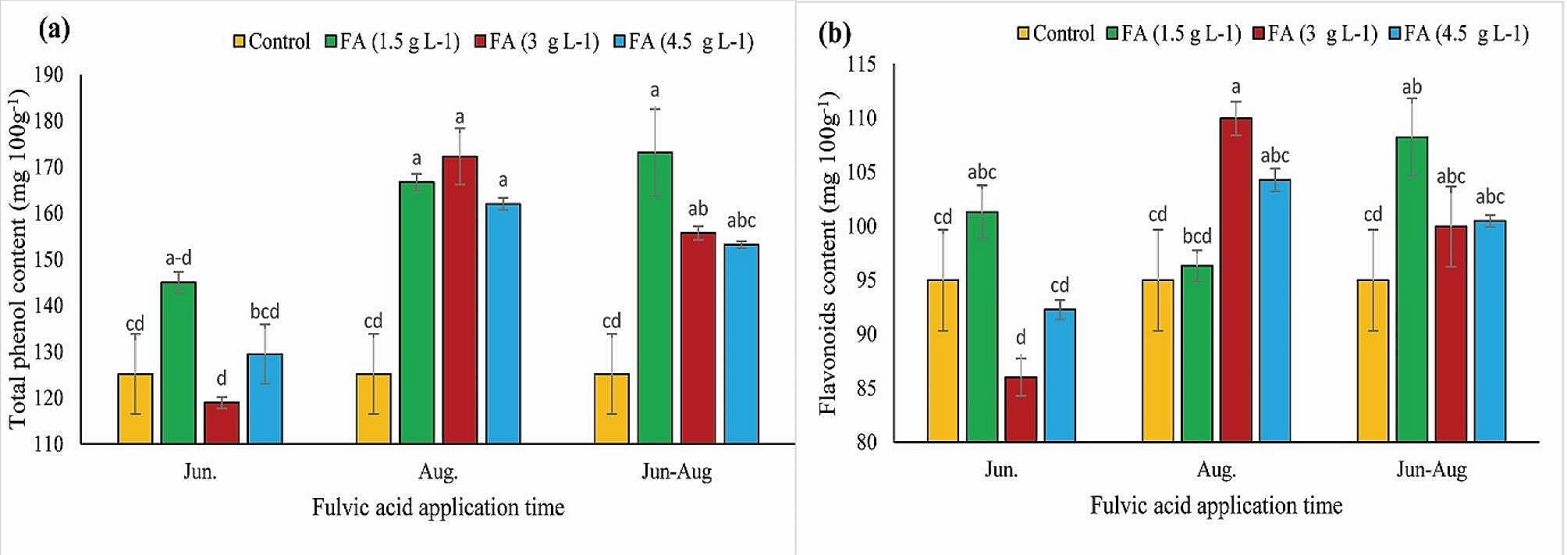
The impacts of different concentrations and application times of fulvic acid on total phenol (**a**) and flavonoids (**b**) content of ‘Kaleh-Ghoochi’ pistachio nut. Different letters show significant differences among means according to the Duncan test at *P* < 0.05

As depicted in Fig. 1(a), the total phenol content of pistachios treated with fulvic acid exceeded that of the control, with the highest amount (161.57 mg 100 g^− 1^) observed in samples treated with 1.5 g L^− 1^ of fulvic acid. Furthermore, a significant effect of treatment time was observed, with the highest total phenol content (156.52 mg 100 g^− 1^) recorded in nuts treated at the end of August.

Figure 1(b) illustrates the fluctuations in flavonoid content in both control and treated nuts. The highest level (109.95 mg 100 g^− 1^) of flavonoid content was found in nuts treated with 3 g L^− 1^ fulvic acid at the end of August, while the lowest amount (85.99 mg 100 g^− 1^) was detected in pistachios treated with 3 g L^− 1^ of fulvic acid at the end of June.

### Antioxidant capacity

Except for nuts treated with 3 g L^− 1^ fulvic acid at the end of June, all treated kernels exhibited higher antioxidant capacity compared with control nuts. Among the treated nuts, the sample treated with 3 g L^− 1^ fulvic acid at the end of August demonstrated the highest level (77.45%) of antioxidant capacity (Table 3).

### CAT activity

In general, CAT activity in treated nuts surpassed that in control nuts, except for those treated with 4.5 g L^− 1^ fulvic acid in late August, which displayed the highest level (15.81 U mg^− 1^ protein) of CAT activity. All kernels treated with 1.5 g L^− 1^ fulvic acid at all three treatment times (late June, late August, and both late June and August) exhibited higher CAT activity compared with other treatments and the control (Table 3).

### APX activity

The activity of APX enzyme in all treatments was higher than in the control, except for the 3 g L^− 1^ fulvic acid treatment applied in late June, which showed the lowest level (14.58 U mg protein) of APX activity. The highest activity (40.12 U mg^− 1^ protein) of APX was observed in nuts treated with 1.5 g L^− 1^ fulvic acid in both late June and August (Table 3).

### POX activity

Table 3 indicates that the application of fulvic acid effectively controlled POX activity, resulting in lower levels of this enzyme in treated nuts compared with control nuts. The highest POX activity level (1.23 U mg^− 1^ protein) was recorded in control nuts, while the lowest level (0.31 U mg^− 1^ protein) was observed in nuts treated with 3 g L^− 1^ fulvic acid at both the end of June and August.

### SOD activity

Fulvic acid-treated nuts demonstrated higher levels of SOD activity compared with control nuts, with the highest activity (242.64 U mg^− 1^ protein) observed in nuts treated with 4.5 g L^− 1^ fulvic acid in late August (Table 3).

**Table 3:** The impacts of different concentrations and application times of fulvic acid on antioxidant capacity and SOD, CAT, APX, and POX activity of ‘Kaleh-Ghoochi’ pistachio nut

Fulvic acid application time	Fulvic acid concentration (g L^− 1^)	Antioxidant capacity (%)	SOD activity	CAT activity	APX activity	POX activity
			(U mg ^− 1^ protein)			
June	0	57.34 ± 4.022 bc	57.35 ± 3.67 b	5.57 ± 0.66 f	19.33 ± 2.02 cd	1.23 ± 0.03 a
	1.5	75.903 ± 4.314 a	178.07 ± 19.52 a	11.34 ± 1.42 bcd	31.03 ± 4.86 ab	0.6 ± 0.01 b
	3	54.546 ± 5.007 c	91.03 ± 25.18 b	7.66 ± 0.57 ef	14.58 ± 2.01 d	1.01 ± 0.05 a
	4.5	76.98 ± 1.072 a	104.72 ± 44.58 b	9.34 ± 1.02 de	30.71 ± 4.14 ab	1.22 ± 0.34 a
August	0	57.34 ± 4.022 bc	57.35 ± 3.67 b	5.57 ± 0.66 f	19.33 ± 2.02 cd	1.23 ± 0.03 a
	1.5	67.106 ± 5.699 abc	184.81 ± 31.97 a	12.77 ± 1.74 bc	32.91 ± 4.04 ab	0.56 ± 0.01 b
	3	77.45 ± 4.302 a	96.74 ± 25.13 b	8.81 ± 0.53 de	19.6 ± 1.42 cd	1.1 ± 0.02 a
	4.5	75.38 ± 7.144 a	242.64 ± 4.81 a	15.81 ± 0.25 a	38.92 ± 4.41 ab	0.47 ± 0.05 b
June-August	0	57.34 ± 4.022 bc	57.35 ± 3.67 b	5.57 ± 0.66 f	19.33 ± 2.02 cd	1.23 ± 0.03 a
	1.5	76.53 ± 1.17 a	240.74 ± 1.98 a	13.38 ± 0.46 ab	40.12 ± 1.34 a	0.4 ± 0.04 b
	3	71.12 ± 1.61 ab	203.7 ± 9.9 a	11.08 ± 0.23 cd	31.82 ± 5.65 ab	0.31 ± 0.008 b
	4.5	69.68 ± 7.17 ab	182.74 ± 0.68 a	9.96 ± 0.96 de	28.07 ± 0.03 bc	1.06 ± 0.01 a

* Different letters show significant differences among means according to the Duncan test at *P* < 0.05. The numbers following ± sign are the standard errors

### Total chlorophyll and carotenoid content

**Fig. 2:**
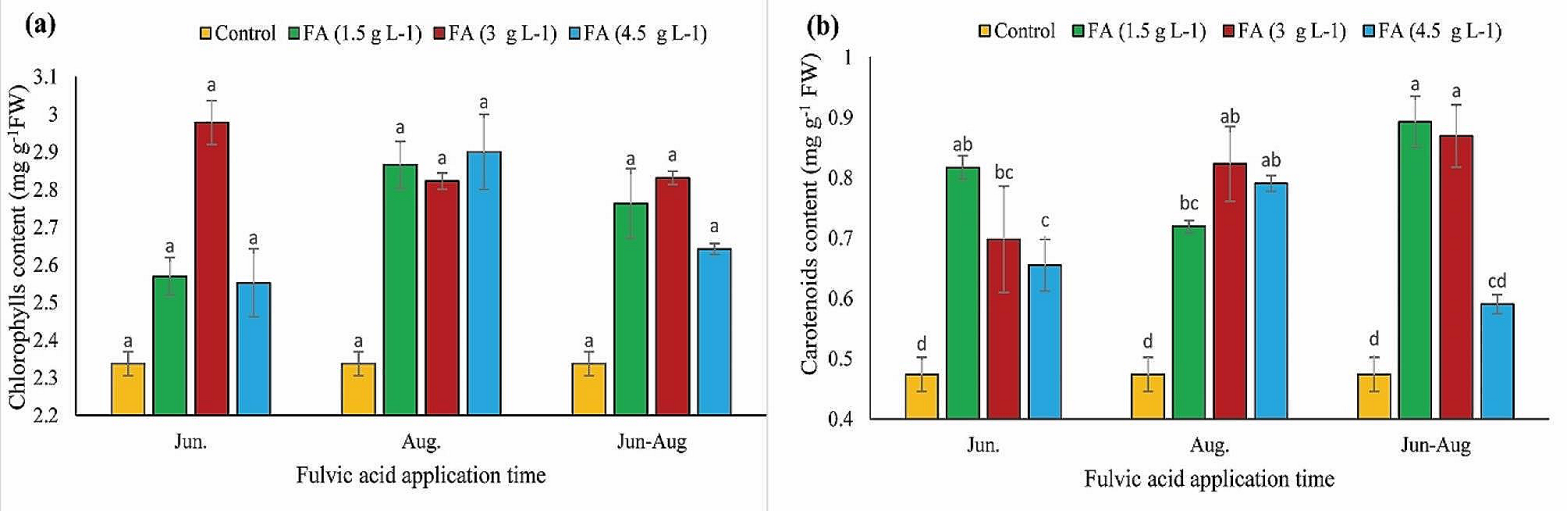
The impacts of different concentrations and application times of fulvic acid on chlorophyll (**a**) and carotenoid (**b**) content of ‘Kaleh-Ghoochi’ pistachio nut. Different letters show significant differences among means according to the Duncan test at *P* < 0.05

According to Fig. 2(a), despite the higher level of total chlorophyll content in fulvic acid-treated nuts in comparison to control nuts, no significant differences were observed between treated and control nuts. The highest level (2.97 mg g^− 1^ FW) of total chlorophyll was observed in nuts treated with 3 g L^− 1^ fulvic acid. In terms of carotenoids, all treated nuts exhibited higher amounts than the control, with the highest content (0.89 mg g^− 1^ FW) belonging to nuts treated with 1.5 g L^− 1^ fulvic acid in both late June and August (Fig. 2(b)).

### K and zn content

**Fig. 3:**
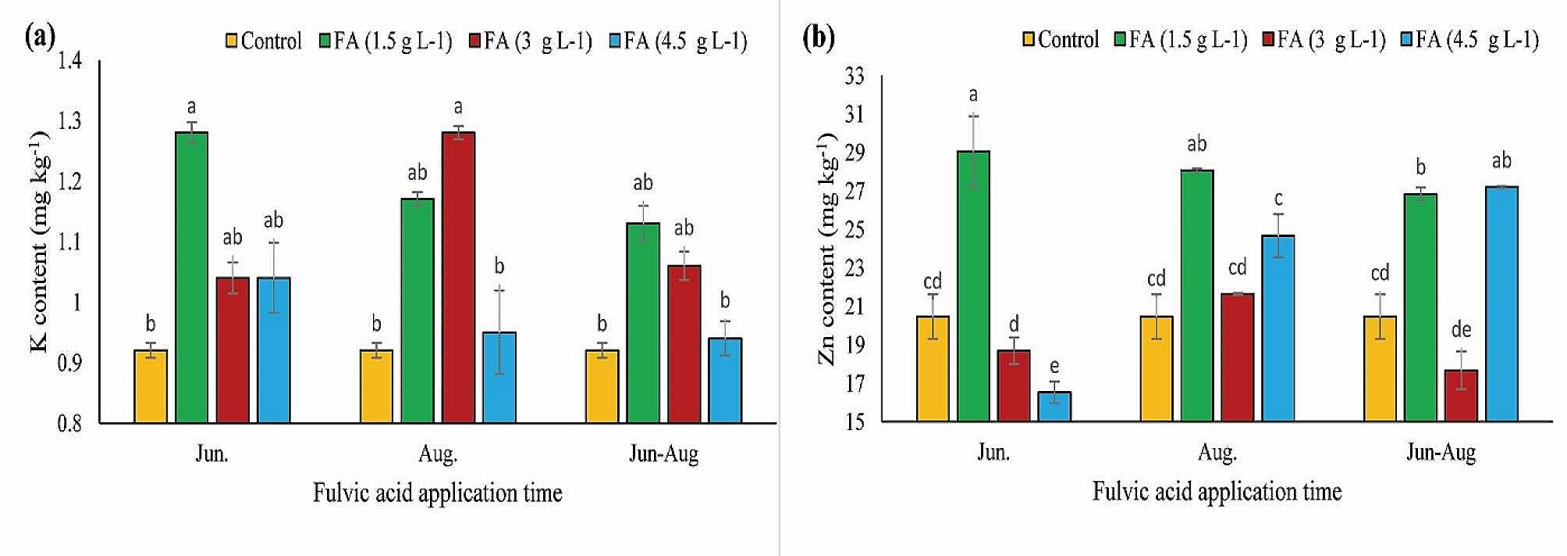
The impacts of different concentrations and application times of fulvic acid on K (**a**) and Zn (**b**) content of ‘Kaleh-Ghoochi’ pistachio nut. Different letters show significant differences among means according to the Duncan test at *P* < 0.05

Figure 3(a) illustrates that the content of K in fulvic-treated nuts was higher compared with control nuts. The highest amount of K was observed in samples treated with 1.5 g L^− 1^ fulvic acid in late June and 3 g L^− 1^ fulvic acid in late August, respectively. In contrast, the lowest amount of K belonged to control nuts. Regarding Zn, except for nuts treated with 3 g L^− 1^ fulvic acid in late June and both late June and August, as well as those treated with 4.5 g L^− 1^ fulvic acid in late June, all other treated nuts showed higher amounts of this mineral content than control nuts. The highest Zn content (29.05 mg kg^− 1^ DW) was reported in nuts treated with 1.5 g L^− 1^ fulvic acid in late June (Fig. 3(b)).

### Soluble carbohydrates and soluble protein content

**Fig. 4:**
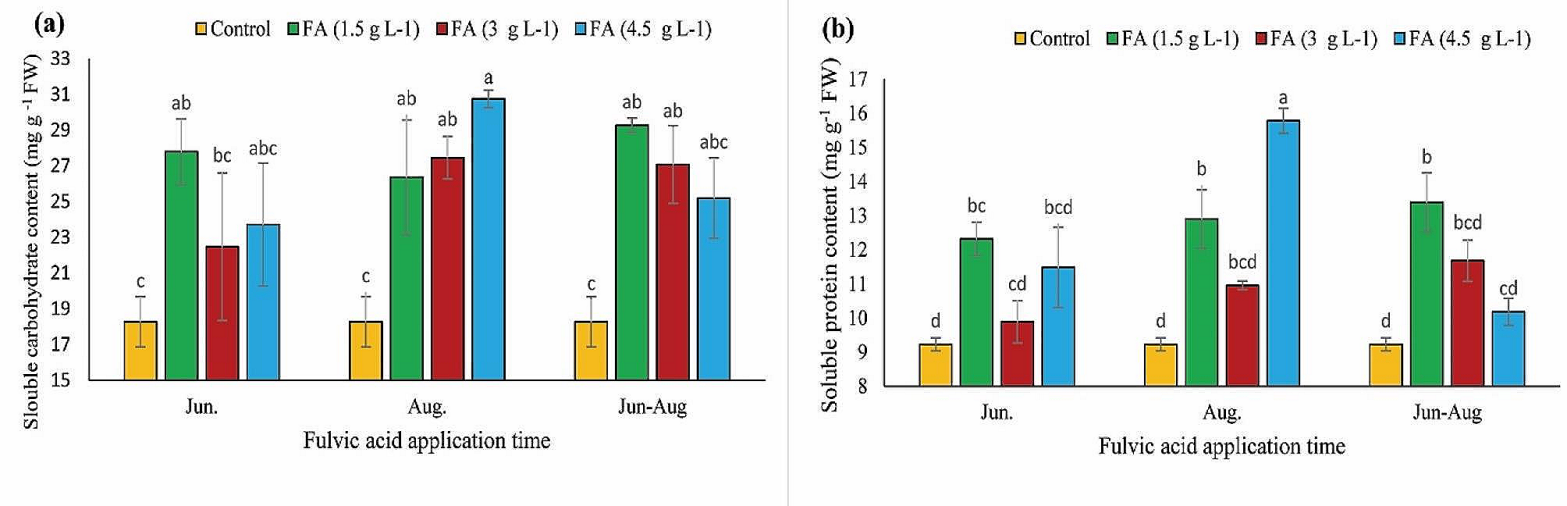
The impacts of different concentrations and application times of fulvic acid on soluble carbohydrate (**a**) and protein (**b**) content of ‘Kaleh-Ghoochi’ pistachio nut. Different letters show significant differences among means according to the Duncan test at *P* < 0.05

The content of soluble carbohydrates in treated nuts exceeded that in control nuts. The nuts treated with 1.5 g L^− 1^ fulvic acid showed the highest amount (27.81 mg g^− 1^ FW) of soluble carbohydrates, while the lowest amount (18.27 mg g^− 1^ FW) was observed in control nuts (Fig. 4(a)). Application of fulvic acid in different concentrations and times enhanced the content of soluble protein, as shown in Fig. 4(b). The highest amount (15.77 mg g^− 1^ FW) of protein was detected in nuts treated with 4.5 g L^− 1^ fulvic acid in late August, while the lowest content (9.23 mg g^− 1^ FW) was observed in control nuts.

## Discussion

In the present study, the application of fulvic acid at various concentrations led to an increase in the total phenol content of pistachio nuts, with 1.5 g L^− 1^ fulvic acid showing the highest efficiency. This finding aligns with studies conducted by Ali et al. [14] on damask rose, Aminifard et al. [26] on pepper, and Asami et al. [27] on strawberries. Plants often face a resource allocation challenge between growth and defense mechanisms, resulting in a competition for precursors between phenolic compounds and proteins [26]. Fulvic acid, as an organic fertilizer, contributes to increased organic and amino acid levels, serving as activators and precursors for growth materials, phytohormones, and secondary compounds in various plants [28]. Under favorable light conditions, the application of fulvic acid promotes carbon enhancement, leading to increased synthesis of C-based secondary compounds such as phenolic compounds [26, 29]. Hence, the study’s results confirm the positive effects of fulvic acid on phenolic compound content reported in other studies.

Humic substances, such as fulvic acid possess the ability to, directly and indirectly, affect the growth and development of plants. The indirect impacts contain the factors which participate in providing energy for helpful organisms, influence the water holding capacity, impact the structure of the soil, distribution of various nutrients from minerals, enhance the trace minerals accessibility, and increase soil’s fertility. The changes which occurred in plant metabolism following the uptake of organic microelements, such as fulvic acid are considered a direct effect that include the entrance of these substances to plant cells and results in some biochemical changes in cytoplasmic components and membranes of cells [30].

The results also indicate that the flavonoid content of fulvic acid-treated nuts surpassed that of control nuts, aligning with studies on strawberries [31] and tomatoes [32]. Organic fertilizers, including fulvic acid, have been reported to increase flavonoid content in bell peppers and tomatoes compared with conventional and mineral fertilizers [33]. Similar findings on yarrow flowers [34] and lemon fruits [35] indicate that fulvic and humic acids positively influence total phenol and flavonoid content. Flavonoid accumulation appears to be correlated with nitrogen availability, suggesting that the nitrogen content in organic fertilizers, such as fulvic acid, significantly influences flavonoid content in plants [31, 36].

Statistical analysis of the data demonstrated that fulvic acid application increased antioxidant activity. These findings are consistent with studies on pepper [26] and wheat [37], highlighting the ability of organic fertilizers to enhance and modify antioxidant capacity. Apart from influencing factors, such as temperature, light, and cultivar, the content of applied humic compositions, such as fulvic acid, significantly affects the antioxidant activity of plants [26, 38]. Moreover, the study suggests that by using organic fertilizers and restricting the application of herbicides, pesticides, and fungicides, plants enhance the production of antioxidant substances to resist pathogen attacks [39]. Thus, the use of fulvic acid appears to have considerable effects on the antioxidant capacity of various plants.

Antioxidant enzymes, essential components of antioxidant activity, play a crucial role in scavenging reactive oxygen species (ROS). In the present study, fulvic acid application enhanced the activity of SOD, CAT, and APX, strengthening the plant protection system against oxidative stresses by preventing the accumulation of ROS such as superoxide radical (O^2.−^), hydrogen peroxide (H_2_O_2_) and hydroxyl radical (OH) in aerobic cells. This enhancement in antioxidant enzyme activity may be associated with the interception of excess energy by photosynthetic pigments [13]. The coordinated activity of these enzymes is necessary for effective ROS destruction and the development of a robust defense system. These observations align with previous research on maize [13] and wheat [11], affirming the positive effects of fulvic acid on antioxidant enzyme activity.

Fulvic acid foliar spray significantly increased chlorophyll and carotenoid content in pistachio nuts. Among various nuts, pistachio is unique in containing chlorophyll and carotenoids in the kernel [40, 41]. Chlorophyll content in unripe green pistachios is initially high and decreases with the ripening process, while zeaxanthin and lutein indicate the content of xanthophyll carotenoids [42]. Accumulation of carotenoids depends on the physiological, biochemical, and genetic characteristics of a plant species and can be influenced by environmental growth agents such as fertility, temperature, and light [26, 43]. Therefore, organic fertilizers, such as fulvic acid are likely to increase the accumulation of chlorophyll and carotenoids. Fulvic acid has been reported to enhance carotenoid content in pepper fruits and increase total chlorophyll content in tomatoes and grapevines [26, 44, 45].

In the present study, the use of fulvic acid led to an increase in K and Zn content of pistachio kernels, consistent with previous findings on eggplant [46], lettuce [47], tomato [45], and yarrow [34]. The positive impact of organic fertilizers, such as fulvic acid, on plant growth by inducing nutrient uptake has been reported in numerous studies [48, 49]. An interaction between organic fertilizers and metabolic and physiological processes has been proposed to explain these positive effects [50]. Fulvic acid stimulates cell permeability, enhances nutritional element uptake, and participates in the regulation of mechanisms involved in stimulating plant growth [51]. Additionally, humic substances in fulvic acid act directly on specific physiological targets, positively influencing signaling and metabolic pathways involved in plant growth and development. An enhanced ion influx, partially attributable to gene H^+^-ATPase transcriptional activation, may result in the production of a favorable electrochemical gradient [52]. Thus, fulvic acid application can enhance plant growth by increasing nutrient uptake.

The results demonstrating an enhancement in carbohydrate content in fulvic acid-treated samples are in line with findings on damask rose [14], pepper [26], and tomato [53]. Total soluble content is known to be correlated with carbohydrate amount, and the positive influence of fulvic acid on total sugar content suggests its ability to enhance carbohydrate content. Fulvic acid, as an organic fertilizer, can positively influence total sugar content, consequently increasing carbohydrate content.

The application of fulvic acid in the present study led to an elevation in soluble protein content in treated pistachio kernels, aligning with similar observations in wheat [11] and maize [13]. The increase in protein content in fulvic acid-treated nuts may be correlated with chlorophyll content and plant growth positively influenced by fulvic acid. Additionally, the use of fulvic acid results in an increase in antioxidant activity (enzymatic and non-enzymatic components), playing a crucial role in preventing protein and photosynthetic pigment degradation by reactive oxygen species [11].

Finding sustainable and effective methods to enhance crop production along with management of the detrimental impacts of environmental factors, are considered the main necessities of modern intensive agriculture. The application of biostimulants, such as humic substances as novel strategies can be helpful to attain that goal. However, the lack of available information about the repeatability and efficiency of their usage in various fruit production industries leads to some limitations in their application. Moreover, the effective ingredients, the mode of action, and the mechanism of effect of these compounds, as well as the cost of their frequent use, should be taken into consideration. Therefore, future studies should focus on finding the right balance between the desired crop cultivar, control of environmental conditions, and management practices, to provide higher efficiency and yield to the producer and higher quality to the consumer.

## Conclusions

The present study highlights the positive impact of the foliar application of fulvic acid, an organic fertilizer, on pistachio quality and nutritional attributes. Notably, the concentration of 1.5 g L^− 1^ applied in late June and August demonstrated optimal effectiveness. Fulvic acid enhanced antioxidant capacity, increased total phenol, flavonoid, chlorophyll, and carotenoid content, and increased the activity of antioxidant enzymes. Furthermore, improvements were observed in K, Zn, soluble carbohydrates, and protein content. Overall, the practical use of fulvic acid as an organic fertilizer can be more beneficial in improving the antioxidant activity and biochemical characteristics of pistachio kernel, therefore, it can be included in the management program of pistachio orchards.

## Data Availability

The data that support the findings of the present study are available from the corresponding author upon reasonable request.

## Declarations

### Statement specifying permissions

For the present study, we acquired permission to study pistachio (*Pistacia vera* L.) issued by the Agricultural and Natural Resources Ministry of Iran.

### Statement on experimental research and field studies on plants

The either cultivated or wild-growing plants sampled comply with relevant institutional, national, and international guidelines and domestic legislation of Iran.
